# Invited Expert Opinion- Bioinformatic and Limitation Directives to Help Adopt Genetic Addiction Risk Screening and Identify Preaddictive Reward Dysregulation: Required Analytic Evidence to Induce Dopamine Homeostatsis

**DOI:** 10.18103/mra.v11i8.4211

**Published:** 2023-09-14

**Authors:** Kenneth Blum, Mark S Gold, Jean Lud Cadet, Marjorie C. Gondre-Lewis, Thomas McLaughlin, Eric R Braverman, Igor Elman, B. Paul Carney, Rene Cortese, Tomilowo Abijo, Debasis Bagchi, John Giordano, Catherine A. Dennen, David Baron, Panayotis K Thanos, Diwanshu Soni, Milan T. Makale, Miles Makale, Kevin T. Murphy, Nicole Jafari, Keerthy Sunder, Foojan Zeine, Mauro Ceccanti, Abdalla Bowirrat, Rajendra D. Badgaiyan

**Affiliations:** 1. The Kenneth Blum Behavioral & Neurogenetic Institute, Austin, TX., USA; 2. Division of Addiction Research & Education, Center for Sports, Exercise & Psychiatry, Western University Health Sciences, Pomona, CA., USA; 3. Institute of Psychology, ELTE Eötvös Loránd University, Budapest, Hungary; 4. Department of Psychiatry, School of Medicine, University of Vermont, Burlington, VT.,USA; 5. Department of Psychiatry, Wright State University Boonshoft School of Medicine and Dayton VA Medical Centre, Dayton, OH, USA; 6. Division of Nutrigenomics Research, TranspliceGen Therapeutics, Inc., Austin, Tx., 78701, USA; 7. Department of Psychiatry, Washington University School of Medicine, St. Louis, MO., USA; 8. Molecular Neuropsychiatry Research Branch, National Institute on Drug Abuse, National Institutes of Health, Bethesda, MD., USA; 9. Neuropsychopharmacology Laboratory, Department of Anatomy, Howard University College of Medicine, Washington, DC., USA; 10. Center for Pain and the Brain (P.A.I.N Group), Department of Anesthesiology, Critical Care & Pain Medicine, Boston Children’s Hospital, Boston, MA., USA; 11. Division Pediatric Neurology, University of Missouri, School of Medicine, Columbia, MO., USA; 12. Department of Child Health – Child Health Research Institute, & Department of Obstetrics, Gynecology and Women’s Health School of Medicine, University of Missouri, MO., USA; 13. Department of Nutrigenomic Research, Victory Nutrition International, Inc., Bonita Springs, FL, USA; 14. Department of Pharmaceutical Sciences, Texas Southern University College of Pharmacy and Health Sciences, Houston, TX, USA; 15. Division of Personalized Mental Illness Treatment & Research, Ketamine Infusion Clinics of South Florida, Pompano Beach, Fl., USA; 16. Department of Family Medicine, Jefferson Health Northeast, Philadelphia, PA, USA; 17. Behavioral Neuropharmacology and Neuroimaging Laboratory on Addictions, Clinical Research Institute on Addictions, Department of Pharmacology and Toxicology, Jacobs School of Medicine and Biosciences, State University of New York at Buffalo, Buffalo, NY 14203, USA; 18. Department of Psychology, State University of New York at Buffalo, Buffalo, NY 14203, USA; 19. College of Osteopathic Medicine of the Pacific, Western University of Health Sciences, Pomona, CA., USA; 20. Department of Radiation Medicine and Applied Sciences, UC San Diego, 3855 Health Sciences Drive, La Jolla, CA 92093-0819, USA; 21. Department of Psychology, UC San Diego, Health Sciences Drive, La Jolla, CA, 92093, USA; 22. Peak Logic, San Diego, CA., USA; 23. Department of Human Development, California State University at long Beach, Long Beach, CA., USA; 24. Division of Personalized Medicine, Cross-Cultural Research and Educational Institute, San Clemente, CA., USA; 25. Department of Psychiatry, Menifee Global Medical Center, Palm Desert, CA., USA; 26. Sunder Foundation, Palm Springs, CA, USA; 27. Awareness Integration Institute, San Clemente, CA., USA; 28. Department of Health Science, California State University at Long Beach, Long Beach, CA., USA; 29. Società Italiana per il Trattamento dell’Alcolismo e le sue Complicanze (SITAC), ASL Roma1, Sapienza University of Rome, Rome, Italy; 30. Department of Molecular Biology and Adelson School of Medicine, Ariel University, Ariel, Israel; 31. Department of Psychiatry, South Texas Veteran Health Care System, Audie L. Murphy Memorial VA Hospital, Long School of Medicine, University of Texas Medical Center, San Antonio, TX., USA; 32. Department of Psychiatry, Mt Sinai University School of Medicine, New York, NY., USA

## Introduction

Addiction, albeit some disbelievers like Mark Lewis [1], is a chronic, relapsing brain disease, resulting in unwanted loss of control over both substance and non- substance behavioral addictions leading to serious adverse consequences [2]. Addiction scientists and clinicians face an incredible challenge in combatting the current opioid and alcohol use disorder (AUD) pandemic throughout the world. Provisional data from the Centers for Disease Control and Prevention (CDC) shows that from July 2021–2022, over 100,000 individuals living in the United States (US) died from a drug overdose, and 77,237 of those deaths were related to opioid use [3]. This number is expected to rise, and according to the US Surgeon General it is highly conceivable that by 2025 approximately 165,000 Americans will die from an opioid overdose. Alcohol abuse, according to data from the World Health Organization (WHO), results in 3 million deaths worldwide every year, which represents 5.3% of all deaths globally [4].

The National Institute on Drug Abuse (NIDA) and the National Institute on Alcohol Abuse and Alcoholism (NIAAA) continue to struggle with the generation of novel approaches to combat the severity of the current substance abuse epidemic. Medication-assisted treatments (MAT) that have been approved by the US Food and Drug Administration (FDA) work primarily by inhibiting dopamine function and release at the pre-neuron in the nucleus accumbens [5–9]. Although MAT has reduced overdose deaths, costs, and health care events, it is pertinent to devise a long-term strategy to return MAT patients to premorbid functioning. Medication-assisted treatments routinely fail [10], and when discontinued, relapse and overdose occur at rates similar to those of untreated patients. Neurologically, MAT may induce persistent changes that compromise endorphin, dopamine, and multiple brain systems. While chronic use of agonist therapies may be necessary in the absence of other options, there is limited data on chronic vs. acute use harm reduction [11,12]. However, there is evidence that treatments themselves, like long-term agonist treatments for opioid use disorder (OUD), may also cause Reward Deficiency Syndrome (RDS) [13], which is a breakdown of reward neurotransmission that causes a broad range of addictive, impulsive, and compulsive behaviors. This can result in harm and fatal consequences that eclipse the size of the current viral COVID-19 epidemic.

Globally, drug overdoses tend to be the highest in the (US), however, it is still a significant international issue that requires urgent and innovative solutions [14,15]. Short-term opioid substitution therapy can decrease harm; however, long-term patients’ risk being locked into a lifetime of substance use disorder (SUD) [16]. On the other hand, inducing “psychological extinction” by weakening a conditioned response over time using the narcotic antagonist, Naltrexone, blocks delta and Mu opioid receptors [17–19]. However, one major difficulty encountered when using narcotic antagonism is patient compliance, which is moderated by the individual’s genetic antecedents [20]. Other FDA-approved therapies to treat alcoholism function through the inhibition of dopaminergic signaling [21,22].

Modification or altered DNA of gene expression has been associated with dependence, withdrawal, and relapse of addictive substance and non-substance- dependent subjects in both animal and human studies [23–27]. A number of studies, especially from Nestler’s lab, revealed numerous expression-altered genes related to substance-dependence such as immediate-early genes, transcription factors, and various neurotransmitter genes [28]. For example, immediate-early genes include members of the Fos family (Fos, FosB), the Jun family (c-Jun, JunB, and JunD) and Zif268 (Egr1) overexpressed transiently or permanently in response to a wide range of addictive psychoactive agents.

Transcription factors (CREB, NF-κb) could exert a crucial role in dependence development by influencing the expression of numerous genes simultaneously [29,30].

Earlier work from Reiter’s laboratory involving darkness induced excessive drinking framed the importance of circadian rhythm revealing the role of melatonin in alcoholism and the pineal gland [31–33]. Certainly, the field is rift with both animal and human studies related to at least seven neurotransmitter systems and receptology especially related to dopamine and NMDA, neurotrophic factors, and CLOCK genes in terms of genetically and neuro-epigenetically induced reward deficiency and all addictive behaviors [34–39].

Obviously, identifying the genes responding to addictive substance exposure and even behavioral addictions [40] and uncovering their regulators should enhance our comprehension of the mechanisms underlying all addictions, and provide a putative gene map for potential therapies for addiction and relapse. With this in mind, Shi et al., utilizing current multi-omic data from multiple studies developed ADDICTGENE (http://159.226.67.237/sun/addictgedb/) [41]. In their study [41] they integrated gene expression, gene-gene interaction, gene-drug interaction and regulatory annotation for over 33,821 items of differentially expressed genes associated with 7 commonly abused substances across three species (human, mouse, rat) from 205 publications. Shi et al [41] suggests that an easy-to-use web interface of Addict Gene which allows users to search and browse multidimensional data on differentially expressed genes (DEGs) of their interest: 1) detailed gene-specific information extracted from the original studies; 2) basic information about the specific gene extracted from NCBI; 3) SNP associated with substance dependence and other psychiatry disorders; 4) expression alteration of specific gene in other psychiatric disorders; 5) expression patterns of interested gene across 31 primary and 54 secondary human tissues; 6) functional annotation of interested gene; 7) epigenetic regulators involved in the alteration of specific genes, including histone modifications and DNA methylation; 8) protein-protein interaction for functional linkage with interested gene; 9) drug-gene interaction for potential draggability. Furthermore, there is robust evidence that cognitive, emotional, and behavioral disturbances observed in psychiatric illnesses, including Reward Deficiency Syndrome (RDS), connect with functional deficits in neurological networks [42–48]. While this eloquent research serves a real purpose for further scientific exploration to uncover molecular mechanisms, it does not provide real clinical usage in terms of early identification of reward dysregulation or RDS [49]. Certainly, behaviors and disorders linked to self-regulation, such as substance use, antisocial behavior, and attention-deficit/hyperactivity disorder, are collectively alluded to as externalizing and have shared genetic liability. While we encourage continued research using large population genome-wide association study (GWAS) studies [50–52], we believe there is an urgent need for an accurate, but not an exhaustive genetic addiction risk severity test for prediction purposes not a diagnostic [53]

## Bioinfomatic Directives

Bioinformatics is an interdisciplinary field that involves software tools for understanding biological data, especially when the data sets are very large and complex. In some cases, bioinformatics includes computer programing like AddictGen [41], and others [53] repeatedly used to help identify candidate genes and singel nucleotide polymorphisms (SNPS). Most importantly, such identification helps scientists to better understand the genetic basis of diseases like RDS, linked to novel adaptations, and even genomic differences between different ethnic populations. For example, it is known that the American Indians carry the Dopamine D2 Receptor (DRD2) A1 allele at 85% compared to the Ashkenazi Jew at only 6 percent [54]. One other important aspect of bioinformatics helps analyze and catalogues specific neurotransmitter pathways and their networks that reflect systems biological approaches enabling the simulation and modeling of DNA and RNA and their interaction. In a more general utilization of the scientific term, historically, bioinformatics did not mean what it means today. In fact, Paul Hogeway and Ben Hesper coined it in 1970 to refer to the study of information processes in all biotic systems [55]. Importantly, for this article we refer to bioinformatics in a more historical way to emphasize several limitations and caveats that must be considered so that the scientific playing field related to the overall concept referred to as “Reward Deficiency Syndrome Solution System” [RDSSS] might generate real scientific retort accompanied with required additional intensive investigation with the goal of ultimate acceptance in the field [56].

With this in mind, we will briefly address a number of elements that currently constitute RDSSS including: 1) a 29-item Reward Deficiency Syndrome Questionnaire (RDSQ29) and validation; 2) GARS: population genomic differences, polygenic scoring vs FDA approved Genetic Health Risk (GHR), GWAS vs. candidate approaches, reductionistic genetic screening at birth, ethical considerations, and the Genetic Information Nondiscriminatino Act (GINA) laws; 3) Induction of homeostasis alternatives: 1) KB220 2) rTMS 3) Brain Stimulation.

## RDS-Q29

RDS integrates psychological, neurological, and genetic factors of addictive, impulsive, and compulsive behaviors. In a recently published article by Kótyuk et al [57] a validation related to the RDSQ29 questionnaire originally developed by our laboratory, was further developed and tested to assess the psychological aspects of RDS. Specifically, data was collected on two college and university samples. Exploratory factor analysis (EFA) and confirmatory factor analysis (CFA) were performed on Sample 1 (N = 1726), and confirmatory analysis was conducted on an independent sample (N = 253). Impulsivity and sensation-seeking were assessed. Based on EFAs, a the RDSQ-29 was developed, containing four subscales (lack of sexual satisfaction, activity, social concerns, and risk-seeking behavior). CFA indicated good fit (comparative fit index (CFI) = 0.941; Tucker-Lewis index (TLI) = 0.933; root mean square error of approximation (RMSEA) = 0.068). Construct validity analysis showed a strong relationship between sensation-seeking and the RDS scale. Kótyuk et al [57] suggested that the RDSQ-29 is an adequate scale assessing psychological and behavioral aspects of RDS. The RDSQ-29 assesses psychological and behavioral characteristics that may contribute to addictions generally. While this does assess the psychological aspects of RDS it does not yield important DNA antecedent polymorphisms. Moreover, future work requires the development of stratification linked to for example “preaddiction” as espoused by McLellan et al. [58]. In seeking an informative scoring of the RDSQ29, we are poised to develop a meaningful trichotomization (mild, moderate, and high) utilizing the precepts of lack of sexual satisfaction, activity, social concerns, and risk-seeking behavior. We believe that when this is accomplished the RDSQ29 would be a valuable tool to help assess preaddiction as discussed in our most recent published article [59]

It is noteworthy that Volkow (Director of NIDA) and Koob (director of NIAAA) are encouraging the psychiatric field to include the concept of “preaddiction” as a new inclusion for the DSM. Relevant to this suggestion is the possibility of developing a test to help categorize mild, moderate, or high risk for future addictive-like behaviors. With this in mind, based on our initial work and now with many other global scientists, the preaddiction classification is best captured with the construct of dopamine dysregulation (net attenuation of function due to the inappropriate or dysregulation involving at least seven major neurotransmitter systems inculding, Serotonergic, Cannabinergic, Opioidergic, GABAergic, Glutaminergic, Acethylcholinergic, and Dopaminergic) or specifically in reward deficiency or net hypodopaminergia at the meso–limbic brain reward circuitry [16].

Currently, there are 1,449 articles listed in PUBMED (11/15/22), whereby north of 47% are independent of our laboratory, and 233 articles listed in PUBMED using the search term “Reward Deficiency Syndrome”. Our point here is that while the term preaddiction resonates well with the historical advance in the diabetic field, scientifically, the real evidence resides in concepts related to brain neurotransmitter deficits or even, in some cases, surfeit (especially in adolescence as a neurodevelopmental event) referred to as “reward dysregulation” [60]. It is noteworthy, as pointed out by McLellan et al. [58], that while the Diagnostic and Statistical Manual of Mental Disorders, Fifth Edition (DSM-5) uses 11 equally weighted symptoms of impaired control to define SUDs along a three-stage severity continuum. The common name addiction is reserved for severe SUD, defined by six or more symptoms, and found in approximately 4% to 5% of adults. Those with mild to moderate SUD (i.e., two to five symptoms) comprise a much larger proportion of the adult population (13%) and thus account for far more substance use–related harm to society than those with severe SUD (i.e., addiction).

However, treatment efforts and public health policies have focused almost exclusively on those with serious, usually chronic addictions, virtually ignoring the much larger population with early stage SUDs.

Although harmful substance misuse and early-stage SUDs can be identified and severity progression monitored, little has been conducted, especially where it is most common, in mainstream healthcare settings. Indeed, neither clinicians nor the public even have a commonly understood name for early-stage SUD.

In this regard, regard we are proposing “Reward Deficiency “(meaning lack of normal function) or “Reward Dysregulation” as a general term that does encompass the nosology of “Preaddiction.” In stating this suggestion, we are cognizant that for public awareness, the latter terminology would be more understood. However, for the DSM, Psychiatrists, and other clinicians, the former seems more parsimonious [61]. With this stated following required research and when we have developed the RDSQ29 to display trichotomization-stratification, this index could then be used to help assess preaddiction as suggested [58].

## Genetic Addiction Risk Severity (GARS)

To develop the Genetic Addiction Risk Severity (GARS) test, the ten reward candidate genes selected included the Dopamine receptors (DRD1, 2, 3, 4); Dopamine Transporter (DAT1); serotonin transporter, COMT, MAO, GABA, Mu opiate receptor and some SNPs and point mutations chosen to reflect a hypo dopaminergic trait. The genes determined to negatively effect the net release of dopamine at the brain reward site were chosen from thousands of association studies providing evidence of the specific risks for all addictions. These alleles were proposed for a GARS panel in case–control studies, specifically for alcoholism (see Table 1) [see ref. 62 for further explanation, permission by Blum K].

In previous research from Blum et al. [63] evaluating 273 mixed-gender patients attending seven treatment centers who completed the Addiction Severity Index (ASI-Media Version V), GARS significantly predicted drug severity (equal or > seven alleles) and alcohol severity (equal or > seven alleles).

In some cases, the risk estimates for one copy of each variant (not all due to the phenomena of heterosis) may have a higher risk than even for individuals who have two copies of a single variant. Since a patient with either one or two copies is managed similarly clinically, the test report provided to the user will have the same interpretation as the test report for both genotypes. While more work needs to be performed, it is crucial to highlight that at this stage, based on dichotomization of the GARS clinical, any combination of these gene associated alleles that reach the level ≤ 4 loads onto risk for drugs and gene associated alleles that reach the level ≤ 7 loads onto risk for AUD [64]. Once this work is completed, it should provide unequivocal evidence for the validity of the selected risk gene-associated alleles. Although we claim that the selection of these candidate genes reflects dopamine dysregulation in the realm of hypodopaminergia, it is important to understand that the end function of dopamine at post synaptic sites in the meso-limbic system is the net result of at least seven neurotransmitter system iterations. Dopamine is not alone and should not be considered alone.

With the forthcoming of GWAS, there has been a burst of very large studies related to genetic polymorphic antecedents to AUD. While others have found evidence for a number of novel clusters of many genes, mostly second messengers, along with the requirement for convergence of these genes to candidates, our approach focuses on finite number of neurotransmitter pathways. We agree that future GWAS studies seem tantamount to unlocking additional candidates for AUD risk, but we believe the current approach has current hieratic value, needing independent confirmation. While GWAS studies utilize very large sample sizes and many SNPs we are not convinced that controls utilized in this sophisticated research reflect RDS free symptomatology, which may prevent true associations between disease and disease ridden controls [65,66].

It is noteworthy, that due to differences between studies (e.g., gender, age, family history, ethnicities, nationalities, comorbidities, the severity of AUD conditions, or unscreened controls), considerable heterogeneity was observed, potentially leading to publication bias (e.g., asymmetric funnel plots) and/or increased false- positive rates (e.g., Type-I error rates). However, not all studies conducted on GARS included all possible caveats in its previous analyses regarding the aforementioned variables. For example, many of the studies did not provide such information to allow us to include or exclude patients with comorbid disorders in the reported samples; this makes it increasingly challenging to assess the effect of the above covariates inducing heterogeneity among studies. Even though the variables such as publication year, study populations, and diagnostic criteria did not seem to be potential sources of heterogeneity, other possible sources of heterogeneity, such as the onset and duration of alcohol addiction and other comorbid conditions or complications, could not be assessed in any meta-analysis. We are very aware that not only AUD, but all addictive behaviors are characterized by its polygenic complex nature impacted by epigenetic effect. It is also well-known that all addictive disorders have multifactorial pathogenesis and is often comorbid with other substance of choice abuse or even neuropsychiatric disorders, which likely share common genetic risk factors in the dopaminergic reward system [67]. Importantly, gene-by-gene or SNP-by-SNP interaction could not be examined due to a lack of studies on other variants that significantly contribute to the liability of complex addictive phenotypes.

Furthermore, in one meta-analysis, performed by Blum et al. [62], only case-control studies were considered, which are certainly more susceptible to sampling bias resulting from the potential differences between alcoholics and control groups than family-based studies, see Gamma et al. [68]. In fact, the inter-rater reliability for selecting reported studies was not assessed, which could also lead to some biases. Most importantly, as repeatedly argued by our group [65], the control groups in most of the studies previously published by others were not individuals randomly selected from the general population whereas cases were selected, and most controls were poorly screened, contributing to the potential bias in wide range of previously published genomic study results. In addition, another reason why selection bias could have occurred is because the majority of the studies were published in English only. We are proposing herein that improved meta-analyses should be conducted using more sophisticated analysis methods for controlling between-study heterogeneity and publication bias as well as RDS-free controls. In this regard, our laboratory is in the midst of developing control data in thousands of highly screened patients to exclude all possible RDS behaviors, which is easily said but hard to accomplish.

Our work has been based on a number of candidate gene methods which were first initiated by the work of Blum and Noble in 1990 [69] as the first confirmed candidate gene to be associated with alcoholism, as well as several other classic candidate gene association studies in terms of accepted methodology [70–73]. It is our opinion that while we are cognizant of the potential pitfalls linked to the candidate gene approach, including ancestry, the candidate approach currently has a clinically relevant outcome and heuristic value. Certainly, the psychiatric genetic field is moving to GWAS instead of candidate gene research [74], but convergence to candidate genes is required to provide meaning with the enormity of the data. One example of this type of GWAS analysis included a proxy-phenotype meta-analysis of Problematic Alcohol Use (PAU), which combined AUD and problematic drinking in 435,563 individuals from European ancestry [75]. They identified 29 independent risk variants, 19 of them novels. PAU was genetically correlated with 138 phenotypes, including substance use and psychiatric traits. In fact, phenome- wide polygenic risk score analysis in an independent biobank sample (BioVU, n = 67,589) confirmed the genetic correlations between PAU and substance use (e.g., cannabis) and psychiatric disorders, reminiscent of RDS [76].

Along similar lines of investigation, a GWAS study analyzing a sample size of 1,2 million subjects involving both tobacco and alcoholism found 566 genetic variants in 406 loci associated with multiple stages of tobacco use (initiation, cessation, and heaviness) as well as alcohol use, with 150 loci evidencing pleiotropic association [77]. However, when convergence was applied, the authors found evidence for the involvement of many systems in tobacco and alcohol use, including genes linked to nicotinic, dopaminergic, and glutamatergic neurotransmission [78]. As mentioned earlier and to reiterate, our concern related to these GWAS, and our subsequent evaluation is that the controls have not been adequately screened to eliminate all reward deficiency symptomatology and associated disorders (i.e., gambling, hoarding, obesity, excess shopping, PTSD, eating disorders, ADHD, etc.).

Of real interest in the entire mental health field is the search for an accurate, gene- based test to identify heritable risk factors for RDS as conducted based on hundreds of published studies about the role of dopamine in addictive behaviors, including risk for drug dependence and compulsive/impulsive behavior disorders. One important driver is to consider the fact that while GWAS can identify many clusters and even convergence to top candidates, the existence of polygenic scoring involving possibly hundreds to even thousands of genes as observed by Shi et al [41] in their unique development ADDICTGEN, is overly complex. However, instead, we developed GARS as a reductionistic way to capture polymorphisms related to high addiction risk. It is noteworthy that one objective of the GARS test was to address the limitations caused by inconsistent results in many case-control behavioral association studies. We believe that many of the limitations are due to the failure of investigators to properly screen controls for drugs, AUD, and RDS behaviors, including nicotine dependence, obesity, pathological gambling, and internet gaming addiction could prevent accurate interpretation of statistical evaluation thereof causing spurious outcomes. One example of accomplishing RDS-free controls is derived from Blum et al. [65,66], which revealed the prevalence of the DRD2 A1 allele in unscreened controls (33.3%) compared to “Super-Controls” (highly screened RDS controls (3.3%) in proband and family). Thus, to provide the best possible statistical analysis, any RDS-related behaviors must be eliminated from the control group to avoid comparing the phenotype to disease-ridden controls.

In summary, unlike one gene-one disease (OGOD), RDS is polygenetic and complex. Even though the genes selected for GARS are not the only ones associated with all addictive behaviors, we decided to focus on specific reward genes, and associated polymorphisms were chosen based on hypodopaminergia [79]. Other genes, such as alcohol metabolism genes (e.g., alcohol dehydrogenase) combined with GARS, may provide an even stronger association in terms of addiction vulnerability. Utilizing genetic risk assessment, with all of the aforementioned caveats, for early identification is important in prophylaxis, especially in adolescence, as evidenced by common brain mapping of addiction [80].

## Population Genomics Issues

We are pointing out that there is a major issue concerning the underpinnings of genetics and associated polymorphic risk alleles in many genomic-based studies [81]. Essentially, this issue involves the disregard for consideration for the role played by ethnic ancestry, differentially displayed in various nationalities and regions. The important thing to ponder herein is to consider the paucity of this work required by clinicians/scientists in comprehending the benefits of genetic testing, especially in underserved populations [82–87]. To provide a snapshot of published articles that have identified primarily reward type of genes and polymorphisms linked to differential resilience and prevalence associated with African Americans and other ethnic groups, for example, just for OUD, see modified Table 2 from [81]

## Disparity in studies of gene variants in Opioid Use Disorder (OUD)

**Figure 1:**
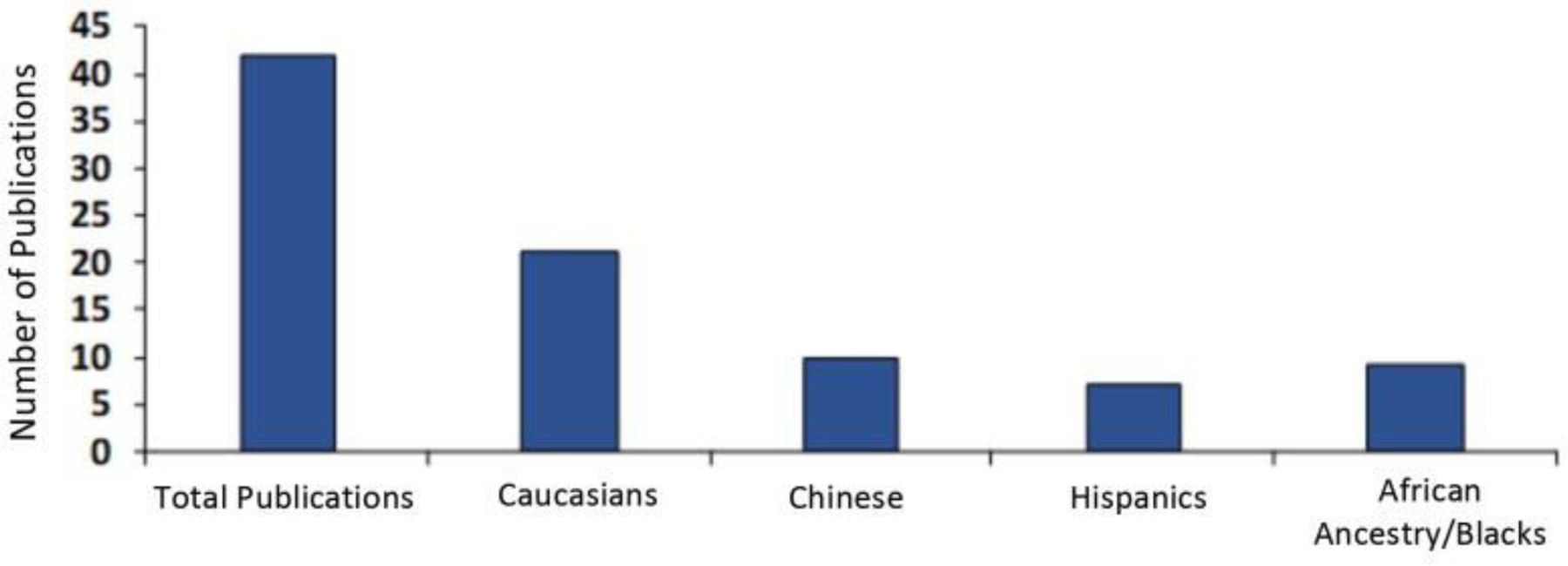
Studies of gene variants and their association with opioid use in different ethnic groups. (permission needed)

Previous and current investigations have shown that OUD may have a genetic basis which may further be complicated by ethnicity and epigenetics. Because of this, it is pertinent that when studying the molecular mechanism underpinning the genetic and even neuroepigenetic basis of OUD, that ethnicity is evaluated. Unfortunately, and a possible cause of spurious results, Hispanics and people of African descent have been ignored in studies emphasizing the genetic basis, for example, OUD. The literature on this subject is summarized in Figure 1.

Importantly, it is well known that individuals of African descent have greater genetic diversity, and as such may have a diverse threshold and efficacy profiles in response to drugs (e.g., psychostimulants) or stimuli [129,130]. Thus, precision addiction medicine is critical for the proper treatment of rewards dysregulation(e.g., OUD) in diverse populations.

In this summary figure 1, Asians are included in exclusive studies from that region. Ironically in America, African American subjects are as highly influenced by the opioid crisis as people of European descent (as a percentage of each population). In fact, according to a recent report by the New York Times, drug deaths, including opioid-related, among blacks in urban counties rose by 41 percent in 2016, exceeding other ethnic groups and especially whites by 19 percent in similar urban communities. This rise continues today. Some genetic testing is available for these genes, especially GARS where many of the genes discussed are tested on a panel to determine risk based on the number of polymorphisms [27,128]. One launderable goal is to develop specialized gene panels that target specific populations to provide a more accurate assessment of any addiction risk as a function of ethnicity.

## Polygenic Scoring Issues

**Figure 2:**
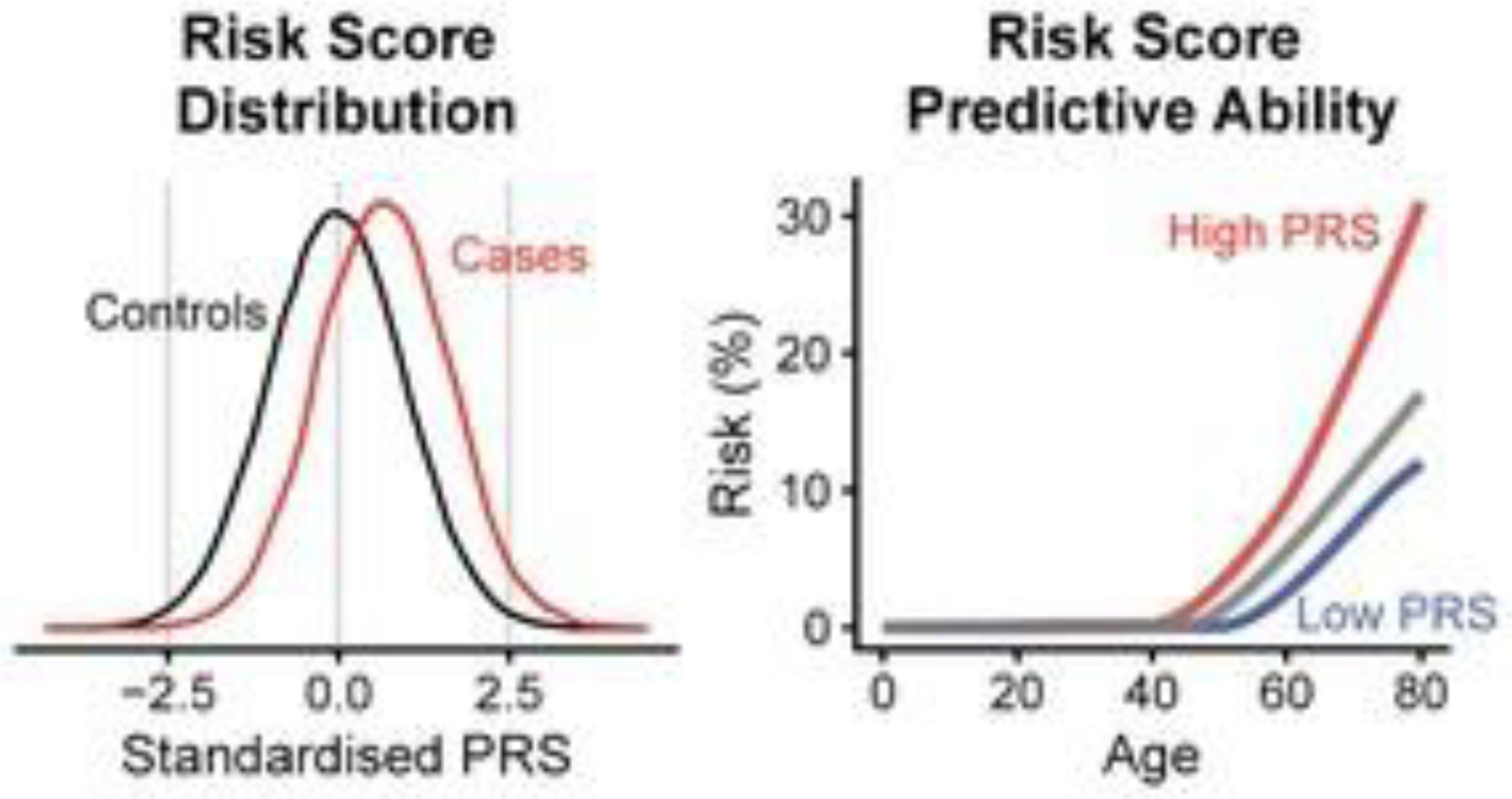
An illustration of the distribution and stratification ability of a polygenic risk score. The left panel shows how in the predictions of disease risk, the PRS on the x-axis can separate cases (i.e., people with the diseases) from the controls (people without the disease). The y-axis describes how many in each group are assigned a certain PRS. To the right, the same population is divided into three groups according to the predicted risk and their assigned PRS. The observed risk is shown on the y-axis, and the separation of the groups is in correspondence with the predicted risks (taken from Wikipedia assessed 11–20-22).

In genetics, a polygenic score (PGS), also called a polygenic risk score (PRS), polygenic index (PGI), genetic risk score, or genome-wide score is a number that summarizes the estimated effect of many genetic variants on an individual’s phenotype, calculated as a weighted sum of trait-associated alleles [130–132]. It reflects one’s estimated genetic predisposition for a given trait and can be used as a predictor for that trait [133,134]. Simply, it provides an estimate of how likely a person is to have a trait only based on genetics, without taking environmental factors into account (e.g., epigenetics). In humans, polygenic scores are typically generated from GWAS data but can also be derived from candidate gene approaches (see Figure 2 for a graphic explanation).

In Psychiatric Genetics, the first confirmed gene associated with severe alcoholism was started by Blum et al. [69]. Current progress in genetics has enabled the development of polygenic predictors of complex human traits, including risk for many important complex diseases like addiction or RDS [135], which are typically affected by many genetic variants that each confer a small effect on overall risk. In a polygenic risk predictor, the lifetime (or age-range) risk for the disease is a numerical function captured by the score, which may, in some cases, depend on the traits of thousands of individual genetic variants (i.e., SNPs). One important benefit of a polygenic score is to provide needed stratification to reflect low, moderate, or high preaddiction trait. In fact, polygenic scores may also empower people to change their lifestyles to reduce risk, for example, overeating [136]. While there is some evidence for behavior modification due to knowing one’s genetic predisposition, additional work from many disciplines is required to assess risk-modifying addictive behaviors. Population-level screening is another use case for polygenic scores and candidate approaches [137]. Most importantly, the goal of population-level screening is to identify patients at high risk for a disease like SUD, who would benefit from an existing therapeutic [138]. Polygenic scores can identify a subset of the population at high risk that could benefit from screening.

**Figure 3:**
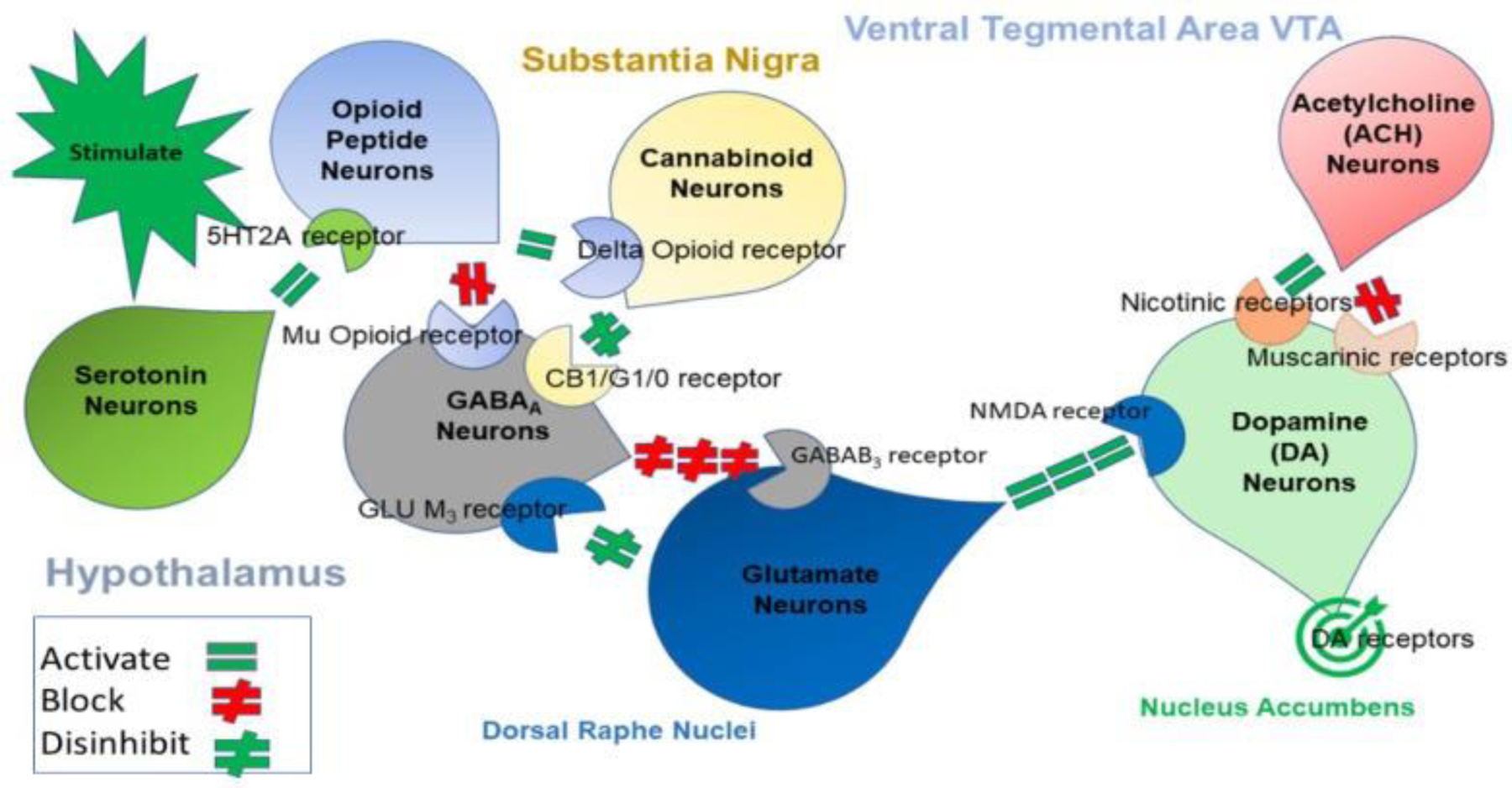
Schematic of the Brain Reward Cascade (BRC) Illustration showing the interaction of at least seven major neurotransmitter pathways in the complex of the Brain Reward Cascade (BRC). In the hypothalamus, environmental stimulation springs the release of serotonin, which in succession via, for example, 5HT-2a receptors activate (equal green sign) the ensuing release of opioid peptides from opioid peptide neurons, also occurring in the hypothalamus. Afterwards, the opioid peptides have, potentially via two different opioid receptors, two distinct effects: one that inhibits (red hash sign) through the mu-opioid receptor (possibly via enkephalin) and projects to the Substantia Nigra to GABAA neurons; or the other, which stimulates (equal green sign) cannabinoid neurons (the Anandamide and 2-archydonoglcerol, for example) via Beta-Endorphin-linked delta receptors, which in turn inhibit GABAA neurons at the Substantia Nigra. Additionally, when activated, cannabinoids, largely 2- archydonoglcerol, may indirectly disinhibit (red hash sign) GABAA neurons through activation of G1/0 coupled to CB1 receptors in the Substantia Nigra. In the Dorsal Raphe Nuclei, glutamate neurons can indirectly disinhibit GABAA neurons in the Substantia Nigra through activation of GLU M3 receptors (red hash sign). GABAA neurons, when stimulated, will, in turn, intensely (red hash signs) inhibit VTA glutaminergic drive via GABAB 3 neurons. It is also feasible that stimulation of ACH neurons at the Nucleus Accumbens ACH will stimulate muscarinic (red hash) or Nicotinic receptors (green hash). Lastly, Glutamate neurons in the VTA will project to dopamine neurons by way of NMDA receptors (equal green sign) to preferentially release dopamine at the Nucleus Accumbens, depicted as a bullseye which indicates a euphoria or “wanting” response. The outcome is that when dopamine release is low (endorphin deficiency), unhappiness is experienced, while general (healthy) happiness is dependent on the dopamine homeostatic tonic set point. (With permission from Blum et al.) [24].

The product development objective for GARS was to develop a genetic-based test that could accurately capture the activity and status of the mesolimbic pathway, known as the “reward pathway.” Therefore, GARS broadly addressess the dopaminergic pathway in the brain known as the Brain Reward Cascade (BRC) and alerts to possible behaviors found to have gene-based associations with hypodopaminergic function (Figure 3).

The search of the scientific literature found polymorphisms of reward genes that associate with risks for RDS behaviors ranging from alcoholism, addiction to opioids, prescription drugs, and non-substance addictions such as comorbid psychiatric conditions, and certain environmental triggers. The specific application of GARS to, for example, preaddiction has been developed [139].

Ten candidate genes and eleven SNPs were selected from the plethora of chemical messengers involved in the neurotransmission of dopamine. The neurotransmission of dopamine follows a systematic interaction of many neurotransmitters and secondary messengers involved in signal transmission across the brain circuitry. Indeed, it is the net release, regulated catabolism, and receptor function of dopamine that is responsible for brain health and impulse control (Figure 3). Dopamine is responsible for feelings of well-being, stress reduction, and other “wanting” behaviors [140,141]. In the original article by Blum et al. [140], they evaluated the hypothesis of “liking” and “wanting ” [142], especially as it relates to RDS, and they found that the incentive salience or “wanting” hypothesis of dopamine function is supported by a majority of the evidence.

A follow-up investigation by File et al. [141] examined the dissociation between “wanting” and “liking” as a function of usage frequency, intensity, and subjective severity in individuals across four substances (alcohol, nicotine, cannabis, and other drugs) and ten behaviors (gambling, overeating, gaming, pornography use, sex, social media use, Internet use, TV-series watching, shopping, and work). Based on their findings using structural equation modeling with 749 participants (503 women, M age = 35.7 years, SD = 11.84) who completed self-report questionnaires, “wanting” increased with the severity, frequency, and intensity of potentially problematic use, while “liking” did not change. Impulsivity positively predicted “wanting,” and “wanting” positively predicted problem uses/behaviors. Reward deficiency positively predicted problem uses/behaviors, and impulsivity and problem uses/behaviors negatively predicted well-being. This kind of data helps the enablement of utilizing psychological based studies [141], to help us understand the real importance of hypodopaminergia and wanting behavior which could impact both seeking substance and non-substance addictive behaviors as self-medicating a compromised brain reward system linked to reduced dopamine function.

For GARS, genes were selected based on their influence on the net release of dopamine at the brain reward site, including DRD1, DRD2, DRD3, DRD4, DAT1, 5-HTTLPR, COMT, MAO-A, GABA, OPRM1. The sequence variants or SNPs, including point-mutations of those genes, were chosen to reflect a hypodopaminergic trait. The basis of the selection was association studies; experimental vs. controls provided strong evidence that specific alleles support a hypodopaminergic trait. The reward genes found by meta-analyses, using PubMed and respected polymorphic alleles are found in Table 1.

After an exhaustive review of the genetic literature related to all RDS behaviors followed by initial testing, only alleles that lead to hypodopaminergia were selected (except for DRD3, see 143). In the review process, we sought to reduce the number of possible genes and alleles and eliminate spurious results. As such, by trial and error, following adding and subtracting genes and alleles, we decided on the proposed 11 allele panel from ten genes. For example, in place of using serotoninergic receptors, serotonin transport was chosen as a way to track serotonin in the synapse, which resulted in an accurate and significant prediction of drug and alcohol severity, linked to a clinical outcome referred to as the ASI-Media version V. This work was a substantial undertaking, involving many alleles, genes, kinases, and second messengers. The use of the BRC, the result of the many years of work done by Blum and Kozlowski [24] and others globally, helped guide our search.

In support, Li and associates [53] found over 800 haplotypes but tracked them to two major pathways, glutaminergic and dopaminergic. This provided a further rationale for the GARS selection criteria. Ten genes and 11 common polymorphisms, including SNPs and Variable Number Tandem Repeats (VNTRs) connected to the promotion of a genetically induced hypodopaminergia, met the final selection for the GARS test. The presence of hypodopaminergia is a complicated but determining condition of the GARS test results based on a polygenic score.

However, the search for studies that report low-dopamine function associated with specific SNPs of reward genes formed the cornerstone of the development of the GARS test. While there are many possible addiction-related genes, as pointed out by Li et al. [53], neurotransmitter pathways located in the mesolimbic/pre-frontal cortices, including the Serotonergic, Cannabinoidergic, Endorphinergic, GABAergic, Glutaminergic, and Dopaminergic are related to brain reward functioning. Any dysfunction of these pathways can result in unwanted dopaminergic dysregulation. Polymorphisms of reward genes that have been correlated with chronic dopamine deficiency and reward-seeking behavior were selected to finalize the genetic panel.

To develop a polygenic score, of the GARS, the initial sample of 393 subjects who provided cheek cells for genotyping, was drawn, from eight geographically diverse treatment centers in the US [144]. The available sample size of 273 (69%) consisted of individuals who had also completed the ASI-MV questionnaire [145]. The alcohol, and drug severity scores in the ASI-MV were determined using a proprietary algorithm developed by Inflexxion. A laboratory located at the Institute for Behavioral Genetics (University of Colorado Boulder) performed standard genotyping for specific polymorphic risk alleles derived from a panel of reward genes. The subjects, participating in the pilot phase of the GARS analysis self-reported their race as White at 88.1% (n = 244) and were 57.8% (n = 160) male. The average age of the of subjects was 35.3 years (S.D. = 13.1, maximum age = 70, minimum age = 18). This study is a statistical analysis that compared a number of risk alleles to the ASI-MV alcohol and drug severity score of each subject.

Among the ASI analysis sample, the number of risk alleles detected ranged from 3 to 15, and the average was 7.97 (S.D. = 2.34) with a median of 8.0. Preliminary examination of the relationship between GARS genotype panel and the Alcohol Risk Severity Score using the Fishers Exact Test revealed a significant predictive relationship (X2 = 8.84, df = 1, p = 0.004 2 tailed) which remained significant after controlling for age [Hardy-Weinberg Equilibrium intact]. Both age and genetic addiction risk scores were predictive of higher alcohol severity scores as assessed with the ASI-MV. In fact, a lower ASI-score predicted a lower GARS score. To account for non-normality in the distribution, drug scores were transformed to (Log10) before analysis of the relationship between the GARS panel and ASI-MV Drugs Risk Severity Score. The relationship between the GARS panel and the Drug Risk Severity Score was found to be similar but less robust than the observation for the Alcohol Risk Severity. Preliminary examination revealed a nominally significant relationship (B = −0.122, t = −1.91, p = 0.057−2 tailed) in this study, following apriori hypothesis of an association of GARS and ASI predictability of risk in which a one-tailed analysis revealed (P=0.028) for the drug severity (greater than four alleles predicted unspecified drug severity risk). The predictive value of GARS was more robust for alcohol risk severity (a score equal or greater than 7) and for drug risk severity (a score equal or greater than 4).

One potential argument against this scoring has to do with the known but unexplained concept of heterosis. [146]. Molecular heterosis occurs when subjects heterozygous for a specific genetic polymorphism show a significantly greater effect (positive heterosis) or lesser effect (negative heterosis) for a quantitative or dichotomous trait than subjects homozygous for either allele. At a molecular level, heterosis appears counter intuitive to the expectation that if the 1 allele of a two-allele polymorphism is associated with a decrease in gene expression, those carrying the 11 genotypes should show the greatest effect, 12 heterozygotes should be intermediate, and 22 homozygotes should show the least effect. According to [146], three explanations for molecular heterosis are proposed. The first is based on an inverted U-shaped response curve in which either too little or too much gene expression is deleterious, with optimal gene expression occurring in 12 heterozygotes. The second proposes a third independent factor causing a hidden stratification of the sample such that in one set of subjects 11 homozygosity was associated with the highest phenotype score, while in the other set, 22 homozygosity was associated with the highest phenotype score. The third explanation suggests greater fitness in 12 heterozygotes because they show a broader range of gene expression than 11 or 22 homozygotes.

Allele-based linkage techniques usually miss heterotic associations. Because up to 50% of association studies show a heterosis effect, this can significantly diminish the power of family-based linkage and association studies, especially with the DRD2 gene [147]. However, when the rules that are appropriate to polygenic inheritance are used, a significant portion of the controversy is resolved [147]. With all this in mind, our team elected to count all alleles from each parent, so homozygotes counted as two even on the same gene. Our thinking along these lines was based on early research by Noble and Blum and associates [148] involving binding characteristics of the DRD2 Taq A1 compared to DRD2 Taq A2 alleles.

Specifically, in a blind experiment, DNA from the cerebral cortex was treated with the restriction endonuclease Taql and probed with a 1.5-kilobase (kb) digest of a clone (lambda hD2G1) of the human DRD2. The binding characteristics (Kd [binding affinity] and Bmax [number of binding sites]) of the DRD2 were determined in the caudate nuclei of these brains using tritiated spiperone as the ligand. The adjusted Kd was significantly lower in alcoholic than in nonalcoholic subjects. In subjects with the A1 allele, in whom a high association with alcoholism was found, the Bmax was significantly reduced compared with the Bmax of subjects with the A2 allele. Moreover, a progressively reduced Bmax was found in subjects with A2/A2, A1/A2, and A1/A1 alleles, with subjects with A2/A2 having the highest mean values, and subjects with A1/A1, the lowest. The polymorphic pattern of the D2 dopamine receptor gene and its differential expression of receptors suggests the involvement of the dopaminergic system in conferring susceptibility to at least one subtype of severe alcoholism, whereby the number of D2 receptors are reduced with a range of 20–40%.

Therefore, understanding the role of heterosis, which could occur in 50% of candidate association studies, whereby the other 50 % of non-heterosis occurs in these same association studies, we opted to count all present alleles regardless of which parent provided the alleles. In the future, when we can actually weigh each allele once we have developed RDS-free controls, the GARS test will be advanced in that it will not rely on a counting procedure.

## Induction of Dopamine Homeostasis Pharmaceuticals and Non-pharmaceutical Alternatives

Our simple proposal to help restore brain neurotransmitter balance in the afflicted individual with a possible pharmacogenomic personalized approach involves the coupling of a genetic-based addiction risk assessment, for example, GARS test, and customized KB220 [149]. Understanding the common neuromodulating aspects of neurotransmission and its disruption via chronic exposure to drugs and behavioral addictions requires a known approach involving “dopamine homeostasis.” While there is an emerging push for the utilization of “psychedelic medicine” [150] in the short term at low doses via patch delivery systems, we further propose that long-term treatment requires induction of “dopamine homeostasis”[151].

However, along these lines of thinking, Bill Wilson’s psychedelic experience, which led to his becoming alcohol-free (but not nicotine-free, which eventually killed him) and the founding of Alcoholics Anonymous, appears to be consistent with the current reexcitement of psychedelic medicine. A 2012 meta-analysis of LSD therapy, albeit a decade ago, was found to be at least as efficacious a treatment using it in the short-term, as anything we currently have today[152].

Moreover, the work of Mash and associates has paved the way to implicate the idea of psychedelics like Ibogaine to treat addiction [153]

Importantly, there have been a number of studies published showing real utility and scientific benefit in terms of identifying both drug and alcohol risk by utilizing objective DNA polymorphic identification rather than just subjective (but still useful) diagnostic surveys, including family history [154]. There are also a number of clinical trials related to a proposed solution to RDS dilemma, and the proposal of induction of “dopamine homeostasis” utilizing DNA-guided pro-dopamine regulation (KB220) [155]. RDS encompasses many mental health disorders, including a wide range of addictions and compulsive and impulsive behaviors. Described as an octopus of behavioral dysfunction [156], RDS refers to abnormal behavior caused by a breakdown of the cascade of reward in neurotransmission due to genetic and epigenetic influences.

The resultant reward neurotransmission deficiencies interfere with the pleasure derived from satisfying powerful human physiological drives. Epigenetic repair may be possible with precision gene-guided therapy using formulations of KB220, a nutraceutical that has demonstrated pro-dopamine regulatory function in animal and human neuroimaging and clinical trials. Recently, large GWAS studies have revealed a significant dopaminergic gene risk polymorphic allele overlap between depressed and schizophrenic cohorts [157]. A large volume of literature has also identified ADHD, PTSD, and spectrum disorders as having the known neurogenetic and psychological underpinnings of RDS [158–160].

Most importantly, it is quite relevant that many peer-reviewed studies, primarily from our group, revealed a remarkable array of neuropharmacological and clinical benefits involving both animal and human experiments [161–212]. While one might evoke the idea of bias because one investigative group undertook most of the published works is quite understandable from a scientific persepctive. However, the initial results involving five decades of research efforts are quite encouraging [213], but more global independent research is required.

Finally, albeit with some potential bias, KB220Z was shown to increase functional connectivity across specific brain regions involved in dopaminergic function. KB220/Z significantly reduces RDS behavioral disorders and relapse in human DUI offenders. Taking a GARS test combined with KB220Z semi-customized nutrigenomic supplement could effectively restore dopamine homeostasis

## Reward Deficiency Syndrome Issues

Alcohol and other substance use disorders share comorbidity with other RDS disorders, i.e., a reduction in dopamine signaling within the reward pathway. To reiterate, RDS is a term that connects addictive, obsessive, compulsive, and impulsive behavioral disorders. An estimated 2 million individuals in the United States have OUD related to prescription opioids. It is estimated that the overall cost of the illegal and legally prescribed opioid crisis exceeds one trillion dollars. Opioid Replacement Therapy (ORT) is the most common treatment for addictions and other RDS disorders. Even after repeated relapses, patients have been repeatedly prescribed the same opioid replacement treatments. A recent JAMA report indicates that non-opioid treatments fare better than chronic opioid treatments [214]. In addition, research demonstrates that over 50 percent of all suicides are related to alcohol or other drug use. In addition to effective fellowship programs and spirituality acceptance, nutrigenomic therapies (e.g., KB220Z) optimize gene expression, rebalance neurotransmitters, and restore neurotransmitter functional connectivity [169].

By proposing RDS as the “true” phenotype, as opposed to utilizing subtypes like SUD or Behavioral Addictions that involve much more measurement error, the recovery landscape may change. Abnormal behaviors involving dopaminergic gene polymorphisms commonly reflect an insufficiency of usual feelings of satisfaction or RDS. RDS occurs as a result of a dysfunction in the “Brain Reward Cascade” (Figure 3), a complex interaction among neurotransmitters (primarily opioidergic and dopaminergic) in the brain reward circuitry [163]. Individuals with a family history of alcohol use disorder or other addictions may be born with a deficiency in the propensity to generate or utilize these neurotransmitters. Prolonged periods of stress and exposure to alcohol or other substances also can lead to a corruption of the brain reward cascade function [215], especially attenuation of endorphinergic synthesis. Blum et al. [216] assessed the possible association of four variants of dopaminergic candidate genes in RDS (DAT1, DRD1, D2DR, and dopamine beta-hydroxylase gene). Blum et al. [216] genotyped an experimental group of 55 subjects obtained from up to five generations of two independent families, with multiple members affected, compared to heavily screened controls (e.g., N = 30 super control subjects for DRD2 gene polymorphisms). Data associated with RDS behaviors were collected on these subjects and 13 deceased family members.

Among the genotyped family members, the DAT1 and the DRD2 TaqA1 alleles were significantly (at least p < 0.015) more often present in the RDS families than controls. For example, 100% of Family A members (N = 32) possessed the TaqA1 allele, while 47.8% of Family B members (11/23) demonstrated expression of the allele. Significant differences were not found between the experimental and control positive rates for the other variants (see 4).

A results of a study by Blum et al. [216] reinforce the putative function of dopaminergic polymorphisms in RDS behaviors, however, the sample size was limited and linkage analysis is necessary. These findings exhibit the importance of a nonspecific RDS endophenotype and explain how assessing single subset behaviors of RDS may produce spurious results. The utilization of a nonspecific “reward” phenotype could be a paradigm shift in future linkage and association studies involving dopaminergic polymorphisms and additional neurotransmitter gene candidates [217]

## Bayes Theorem and at Birth Predictability to RDS

In probability theory and statistics, Bayes Theorem defines the probability of an event based on previous knowledge of conditions that could be related to the event. Bayes’ theorem refers to Reverend Thomas Bayes (1701–1761), who first utilized conditional probability to establish an algorithm (his Proposition 9) that uses evidence to calculate limits on an unknown parameter, published as “An essay towards solving a problem in the Doctrine of Chances” [218]. In what he called a scholium, Bayes extended his algorithm to any unknown prior cause. Independently of Bayes, Pierre–Simon Laplace, in 1774 and later in his 1812 “Théorie Analytique Des Probabilités,” utilized conditional probability to formulate the relation of an updated posterior probability from a prior probability, given evidence. Sr Harold Jeffreys put Bayes’ algorithm and Laplace’s formulation on an axiomatic basis. Jeffreys wrote that Bayes’ theorem “is to the theory of probability what the Pythagorean theorem is to geometry.” Blum et al. [219] used this mathematically based theorem to predict the chance that if you carry the DRD2 A1 allele at birth: what is the Predictive Value (P.V.) that the individual would potentially indulge in drug and non-drug behavioral addictive behaviors (RDS)?

The dopaminergic system, particularly the DRD2, has been profoundly implicated in reward mechanisms in the mesolimbic circuitry of the brain. Dysfunction of the D2 dopamine receptors contributes to an aberrant substance-seeking behavior (i.e., alcohol, drug, tobacco, and food). Decades of research indicate that genetics plays an important role in vulnerability to severe substance-seeking behavior. Blum et al. [220] and Archer et al. [221] proposed that variants of the DRD2 are important common genetic determinants in predicting compulsive disease. Blum et al. [221] determined through the Bayes approach that when they added up many RDS behaviors and applied the Predictive Value (P.V.), they found a 74.4% value. This leads to the unfortunate fact that a newborn with the DRD2 variant (A1 compared to A2 [usual]) will have a 74 % chance of developing RDS behaviors and could shift to addiction. In this regard, the full GARS panel (to be explained below) has not yet been analyzed using Bayes Theorem, but we are very confident that the P.V. would even be higher. One caveat with regard to our Bayes approach is that we performed this calculation in the mid-nineties at infancy concerning psychiatric genetics, especially on the heels of our laboratory coining the term “Reward Deficiency Syndrome aka RDS.”

In terms of the Bayes P.V. value of 74.4%, we believe that once RDS-free controls could be developed as well as utilizing the most recent data on reward deficiency should result in a higher P.V. statistic. Unfortunately, this is an alarming unwanted predictability of those children that present a risk for future RDS behaviors. While this is somewhat daunting, it could increase both animal and human studies required to obtain approval for both RDS-Q29 and GARS as tools to help with the “preaddiction” stratification enabling early-on innovative interventions [222]. In fact, Stockings et al. [223] suggested that to prevent SUD and reduce harm, special focus is required to provide evidence on the effectiveness of prevention. Along this line, they [223] encourage taxation, public consumption bans, advertising restrictions, and minimum legal age are all effective measures to reduce alcohol and tobacco use but are not available to target illicit drugs. Specifically, they espouse the fact that social norms and brief interventions to reduce substance use in young people do not have strong evidence of effectiveness. However, roadside psychoactive drug testing and interventions to attenuate injection-related harms have a moderate-to-large effect, but additional research with young people is parsimonious.

Although the molecular mechanisms of RDS are phenomenological, the classification of its appearance is incomplete. The unified definitions of the psychological and behavioral appearance of RDS are unavailable. The proposed RDS includes a set of psychological “symptoms” that can signal its presence. Blum and colleagues refer to the phenomenological and behavioral aspects of the RDS as “an inability to derive reward from ordinary, everyday activities” [224,225]. Dopamine, along with additional reward neurotransmitters, are portrayed as producing this sense of well-being. Individuals with neurotransmitter dysregulation strongly engage in substance-seeking and craving behavior and employ other common hedonic mechanisms to decrease negative emotion [226].

As per the RDS model, insufficiencies in dopaminergic systems leave individuals susceptible to addictive behaviors via the stimulation of the mesolimbic system. Based on the phenomenological appearance of RDS, the purportedly linked disorders and behaviors, and the proposed involvement of the mesolimbic system, RDS would theoretically show relatedness to risk-taking personality traits, such as impulsivity and novelty seeking, as well as mood characteristics, such as anhedonia or depression. The multi-level model of RDS describes a so-called “hypodopaminergic trait,” which associates with psychological dimensions of addictions and potentially addictive behaviors and proposes a specific molecular mechanism. The exception may be most adolescents because of developmental epigenetics, which may induce a hyperdopaminergic state [227].

Despite the promise of the RDS model, some of the proposed associations have received mixed support, and the model needs further empirical testing. For example, as noted above, one of the initial premises of the RDS based on early findings, the relationship between DRD2 variants and addictions, have either been questioned [228] or has shown small effect sizes [225]. Others found that the A1 allele does not increase the risk for alcoholism per se but may be involved in related traits or characteristics [226–236].

Inconsistent association results involving DRD2 variants and addictions suggest more complex etiologies for addictions. These questions were first raised over 20 years ago [226–235], but currently, there is general agreement that addictions are polygenetic and that the DRD2 variants represent a major polymorphic allelic concern [235]. In fact, Nutt et al. [236] correctly suggested that for several decades, addiction has come to be viewed as a disorder of the dopamine neurotransmitter system; however, this view has not led to new treatments. Moreover, they also wrongly suggest that there is robust evidence that stimulants increase striatal dopamine levels and evidence that alcohol may have such an effect, but little evidence, if any, that cannabis and opiates increase dopamine levels. This is not in agreement at all with the current literature [237–241].

Empirical studies also question the link between RDS and food addiction. Benton and Young [242] conducted a meta-analysis of BMI and DRD2 variants to test the hypothesis stating that, similar to SUD in food addiction, the A1 allele is associated with lower levels of DRD2 genes [243,246]. This meta-analysis of 33 studies found no associations for BMI, which they criticized as a definitive measure of food addiction [245]. They concluded wrongly that this meta-analysis did not support the RDS model of obesity or food addiction. However, many of the studies assessed in this meta-analysis suffered from inappropriate food addiction severity phenotypes and a lack of stratification among racial groups. These referenced studies conversely show the involvement of dopamine genetics in food-seeking [246–268].

Furthermore, Nutt et al. [236] made a strong argument for the fact that despite the focus on dopaminergic function and potential anti-addiction treatments based on targeting dopamine, no new treatments have been developed. This position is well understood, but the reason for this failure is not. On a theoretical level, Dackis and Gold [269] pioneered the incorporation of a D2 agonist like bromocriptine to treat cocaine dependence, but because of the powerful effect, and chronic induced downregulation on D2 receptors, this drug was ineffective. It is well known chronic incorporation of this and other D2 agonists induce an unwanted down-regulation of DRD2 [270]. It is also true that the current FDA-approved treatments, for example, alcoholism is based on blocking dopamine function, inducing an anti-reward state [17,271–273].

## Gene Testing at Birth

To suggest that children, even at birth, should be screened for potential RDS (e.g., ADHD) risk alleles may seem too bold and premature. It may, however, be intelligent to at least explore the possibility in the future. In this regard, Bill Moyers of PBS has done some excellent work investigating the plight of future America, suggesting that we should diagnose ADHD very early in life (if not at birth) and couple diagnosis with a safe side-effect-free treatment. State newborn screening tests are performed within the first few days of life to screen for serious, life-threatening diseases. Every baby born in every US state is tested, even if the baby seems healthy and has no symptoms of health problems. State laws mandate that babies be tested between 2 and 7 days of age. Recessive diseases usually occur when both healthy parents naively carry a gene for a recessive disorder, and both pass the gene to their baby. The baby who inherits two copies of the recessive gene is born with this condition except in cases of heterosis. The resulting diseases are often treatable with special diets and/or medications. Early detection of these diseases can prevent mental retardation, other disabilities, and mortality. Pediatric metabolic specialists and nutritionists are required for conditions that necessitate specified diets, like phenylketonuria (PKU) and galactosemia. Parents require education regarding appropriate foods and blood and urine monitoring to ensure that the infant remains unharmed by the disease. Could this same level of expertise be adopted in testing for and treating infants with preaddiction risk predisposition, as well?

## Genetic Testing and Screening

Human medical genetics deals with the role of genes in illness. Traditional analysis of the genetic contribution to human characteristics and illness has involved three types of disorders: 1) disorders due to changes in single genes; 2) polygenic disorders influenced by > 1 gene; and 3) chromosomal disorders. Genetic screening [275] differs from genetic testing. Although the terms are used interchangeably, genetic screening is carried out on a defined (by age, sex, or other risk factors) section or subgroup of the population, in which certain disabilities may be the result of genetic factors. Genetic screening has been defined as: “… a search in a population to identify individuals who may have, or be susceptible to, a serious genetic disease, or who, though not at risk themselves, as gene carriers may be at risk of having children with that genetic disease.”[276]. On the other hand, genetic testing has been defined as: “… the analysis of a specific gene, its product or function, or other DNA and chromosome analysis, to detect or exclude an alteration likely to be associated with a genetic disorder,” and results in a definitive diagnosis for the individual involved [275,276].

Screening programs are crucial in public health care systems where they can identify individuals at serious risk and prevent morbidity by timely treatment. In this regard, the goals are: 1) to improve the health of persons with genetic disorders; 2) to facilitate informed choices regarding reproduction for the carriers of abnormal genes; 3) to alleviate the concerns of families and communities about serious genetic disease: and 4) reduce public health costs. For those institutions seeking to reduce cost and better manage their public health exposure, genetic screening is a good option. There are some concerns that genetic testing of the human population could slide into eugenics.

Eugenics was a social movement that sought to improve the genetic features of human populations through sterilization and selective breeding (for example, sterilization of the mentally “unfit” practiced in some states until the 1970s) [276]. This is not the case for genetic screening and testing for the ADHD phenotype, suggested in order to facilitate early and accurate diagnosis and preventive treatment [277–279]. Nonetheless, it is noteworthy that the negative impacts of genetic screening have ethical implications that can be separated into personal and societal categories of harm.

Personal harm concerns the psychological well-being of the individual and may include increased personal anxiety about labeling, health, and decisions related to infant and prenatal testing. Societal harm, perhaps with more powerful ethical considerations, involves the interaction of society with the individual, with regard to employment prospects, access to health insurance, life insurance, and other benefits, as well as eugenics.

Many ethical issues will need to be confronted following the advent of psychiatric genetics. As knowledge grows regarding the genetic basis of psychiatric disorders, the accepted etiology of most psychiatric disorders will be that environmental factors (epigenetics) interact with multiple predisposing genes. As tests for the genes involved have become more readily available for screening in adults, children, and for prenatal testing, aside from using genetic screening to diagnose predisposition and design treatment for psychiatric illnesses, pressures to use such testing for premarital screening and selection of potential adoptees may develop.

Challenges of genetic testing include the impact that such knowledge can have on the individual, on one’s sense of self; misunderstanding of the consequences of genetic predisposition and discrimination; and using genetic information to deny individals access to, for example, employment and insurance. Most states have some legislation aimed at preventing discrimination. However, coverage by most state laws is spotty.

With the establishment of GINA in the US in 2008, individuals are protected by federal law. Physicians may find that they have new duties created by reports of genetic test results, including addressing common misunderstandings of the consequences of possessing an affected allele and alerting third parties who may share the patient’s genetic endowment.

Some questions about the appropriate disclosure of information to individuals and their family members during the process of genetic research have risen. Germane information about the genes that are being studied, how the subjects of the research are defined, and how information is collected from the proband’s family members should be addressed. In the near-term, medical professionals will need to attend to and resolve these dilemmas, as neglecting them will leave others to make rules to control medical psychiatric practice, including psychiatric genetic research [280].

## Conclusion

It is generally accepted that balancing the brain reward circuit or achievement of “dopamine homeostasis” is a laudable goal instead of inhibiting natural dopamine or prescribing a potent opioid to treat opioid addiction [281]. We are encouraging both the neuroscience and clinical science communities to potentially embrace this disruptive technology with a futuristic view of addressing the notion of what constitutes “standard of care” in the face of the ongoing addiction (alcohol, opioid, psychostimulant, food, etc.) pandemic [282].

While additional research is needed, it is pertinent to begin establishing guidelines that incorporate the knowledge of RDS as an umbrella term for all addictive behaviors. Comprehending neurogenetics and using a systems biological approach (PBM), as previously stated, appears to be the most prudent and marks a breakthrough in restoring joy to the billions suffering globally, particularly in terms of early detection of preaddiction.

## Figures and Tables

**Figure 4:**
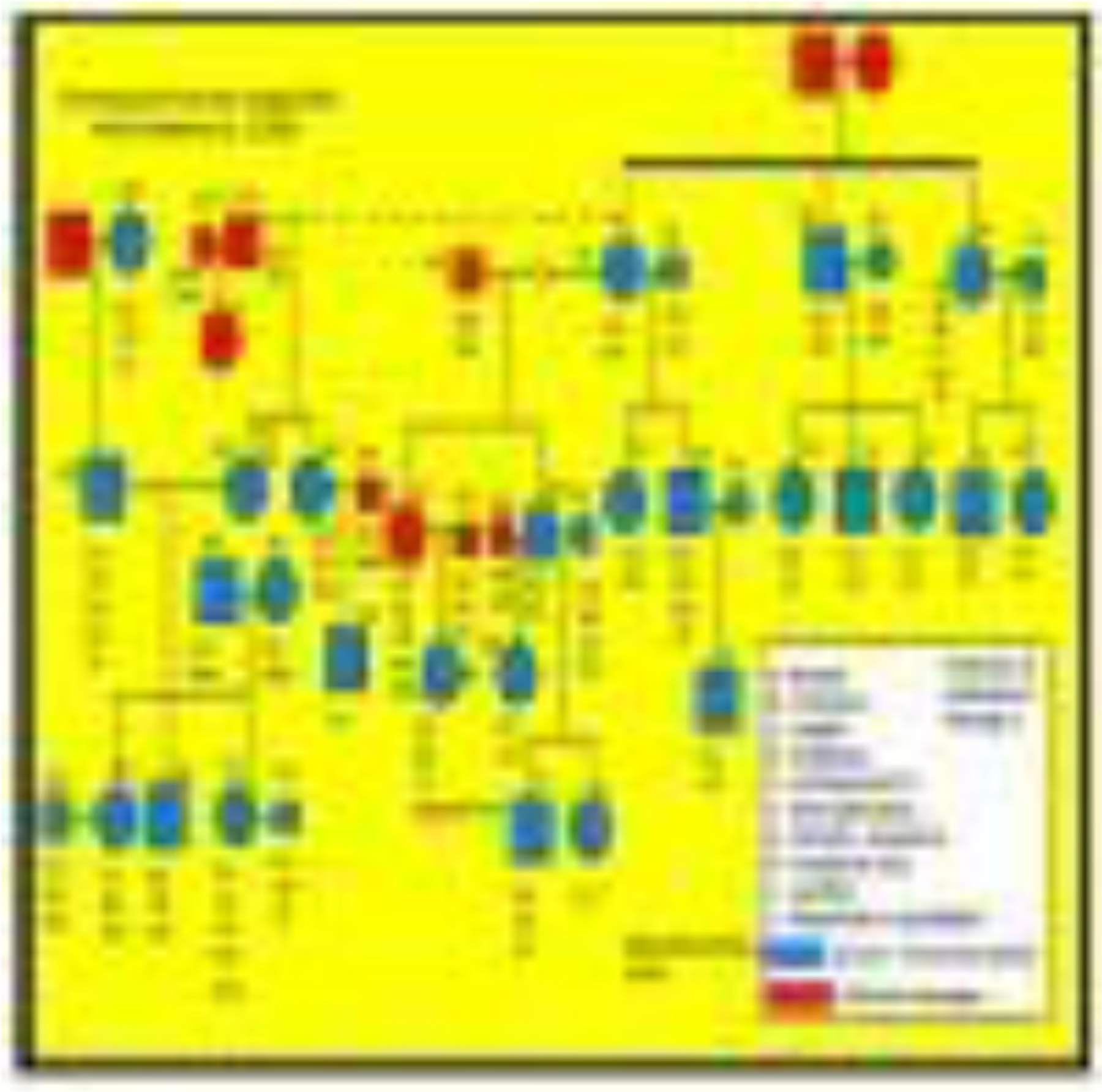
Genotypic results of the Dopamine D2 receptor gene (DRD2) polymorphism of family A (N = 32) corresponded with multiple Reward Deficiency Syndrome (RDS) behaviors [216].

**Table 1.** Gene polymorphisms under consideration and the literature summary.

Gene/Polymorphism	Number ofStudies	Overall Summary
Dopamine D1 Receptor (DRD1): rs4532—risk allele G	3	Several studies supported that genetic variation in DRD1 may influence genetic predisposition to alcoholism. A statistically significant association of DRD1 rs4532 polymorphism with alcohol dependence was found among Indian males (90 cases vs. 122 controls). Other studies also demonstrated that this could be associated with the impulsivity and aggression of AUD patients.
Dopamine D2 Receptor (DRD2): rs1800497—risk allele A1	118	The DRD2 rs1800497 was found to be associated with the risk of AUD and several AUD-related conditions in a meta-analysis of numerous case–control studies (total of 18,290 cases vs. 19,809 controls, including US Caucasian, native and African American, British, French, Italian, Swedish, Finnish, Spanish, Mexican, Brazilian, Scandinavian, and Japanese) pooled with the random effect models.
Dopamine D3 Receptor (DRD3): rs6280—risk allele C (Ser9Gly)	3	Several case–control studies investigated the association between the DRD3 rs6280 polymorphism and alcohol dependence. In a Korean study (243 cases vs. 130 controls), the DRD3 rs6280 polymorphism was significantly associated with AUD development.
Dopamine D4 Receptor (DRD4): rs1800955—risk allele C (48bp repeat VNTR)	35	The DRD4 rs1800955 polymorphism was found to be associated with the risk of developing AUD and AUD-related conditions in a meta-analysis of various case–control studies (total of 2997 cases vs. 2588 controls, including US Caucasian, Mexican American, and Indian) pooled with the random effect models.
Dopamine Transporter Receptor (DAT1): SLC6A3 3’-UTR—risk allele A9 (40bp repeat VNTR)	43	The central dopaminergic reward pathway is likely involved in alcohol intake and the progression of alcohol dependence. DAT1 is a principal regulator of dopaminergic neurotransmission. From the meta-analysis of numerous case–control studies (total of 3790 cases vs. 3446 controls) pooled with the random effect models, the DAT1 SLC6A3 3’-UTR risk allele was found to be marginally associated with the risk of AUD and AUD-related conditions.
Catechol-O-Methyl-transferase (COMT): rs4680—risk allele G (Val158Met)	13	COMT is a strong candidate gene that contributes to SUD and schizophrenia. A meta-analysis of several case–control studies (total of 1212 cases vs. 933 controls, including US Caucasian, Finnish, Croatian, and Taiwanese) pooled with a random effect model, the association of COMPT rs4680 polymorphism with the risk of developing AUD and AUD-related conditions was found with marginal statistical significance.
μ-Opioid Receptor (OPRM1): rs1799971—risk allele G (A118G)	28	Opioid receptors play an essential role in ethanol reinforcement and alcohol dependence risk. Polymorphisms in the OPRM1 gene expressing μ-opioid receptors could be significantly associated with some features of alcohol dependence. From the meta-analysis of case–control studies (total of 3096 cases vs. 2896 controls, including US Caucasian, Spanish, Turkish, and Asian), pooled with the random effect model, the results indicated that a functional OPRM variant is associated with the risk of alcohol dependence with marginal statistical significance.
γ-Aminobutyric Acid (GABA) A Receptor, β−3 Subunit (GABRB3): CA repeat—risk allele 181	6	The GABAergic system has been implicated in alcohol-related behaviors. From case–control studies (171 cases vs. 45 controls), the association of variants of the GABRB3 gene with alcohol dependence is, however, inconclusive. A more extensive controlled study is required for improved results.
Monoamine Oxidase A (MAO-A): 3’ 30bp VNTR -risk allele 4R DNRP	6	The function of monoamine oxidase (MAO) in alcoholism was determined using several case–control studies (170 cases vs. 177 controls). Although genetic heterogeneity is suspected of underlying alcoholism and MAO-A mutations may play a role in susceptibility to alcoholism, the overall results were not found to be statistically significant. A more extensive controlled study is required to obtain conclusive results.
Serotonin Transporter Receptor (5HTT) Linked Promoter Region (5HTTLPR) in SLC6A4: rs25531—risk allele S’	20	Serotonin (5-HT) has been demonstrated to regulate alcohol consumption. Since the activity of the 5-HT transporter protein (5-HTT) regulates 5-HT levels, it may contribute to the risk of alcohol dependence. A meta-analysis of case–control studies (total 9996 cases vs. 9950 controls) pooled with the random effect models showed a significant association between alcohol dependence and the serotonin-transporter-linked promoter region (5-HTTLPR), which is a polymorphic region in the SLC6A4 gene.

**Table 2:** Publications on gene variants associated with opioid use disorder (OUD) as related to diversity of study cohort.

Genes	Variants (SNPs and Haplotypes)	Publication Describing Association with Opioid Dependence	Publications that Consider Caucasians	Publications that Consider Chinese	Publications that Consider Hispanics	Publications that Consider African Ancestry
DRD2	rs6275; rs6277; rs1076560; rs1799978; rs1800496; rs1801028	Clarke, T.K. et al. [88]; Hou, Q.F. and Li, S.B. [89]; Vereczkei, A. et al. [90]; Lawford, B.R. et al. [91].	Clarke, T.K. et al. [88]; Vereczkei, A. et al. [90]; Lawford, B.R. et al. [91].	Hou, Q.F. and Li, S.B. [89].		Clarke, T.K. et al. [88].
DRD3	rs6280 rs9825563 rs2654754 rs9288993 rs1486009	Levran, O. et al. [92]; Kuo, S.C. et al. [93].	Levran, O. et al. [92].	Kuo, S.C. et al. [93].		
DRD4	rs1800955	Vereczkei, A. et al. [90]; Szilagyi, A. et al. [94]; Ho. A.M. et al. [95]; Lai, J.H. et al. [96].	Vereczkei, A. et al. [90]; Szilagyi, A. et al. [94].	Ho. A.M. et al. [95]; Lai, Lai J.H. et al. [96].		
OPRM1	rs1799971 rs1799972	Bond, C. et al. [97]; Crowley, J.J. et al. [98]; Szeto, C.Y. et al.	Bond, C. et al. [97]; Crowley, J.J. et al. [98]; Hastie, B.A. et al.	Szeto, C.Y. et al. [99]; Shi, J. et al. [100].	Bond, C. et al. [97]; Crowley, J.J. et al. [98]; Hastie,	Bond, C. et al. [97]; Crowley, J.J. et al. [98]; Hastie, B.A. et al. [101].
OPRD1	rs1042114 rs678849 rs10753331 rs529520 rs581111 rs2234918	Zhang, H. et al. [102]; Levran, O. et al. [103]; Nelson, E.C. et al. [104]; Levran, O. et al. [105]; Crist, R.C. et al. [106]; Sharafshah, A. et al. [107]; Beer, B. et al. [108].	Zhang, H. et al. [102]; Crist, R.C. et al. [109];Levran, O. et al. [103]; Nelson, E.C. et al. [104]; Beer, B. et al. [108].			Levran, O. et al. [105]; Crist, R.C. et al. [106].
KCNC1; KCNG2	rs60349741 rs62103177	Gelernter, J. et al. [109].	Gelernter, J. et al. [109].			Gelernter, J. et al. [109].
OPRK1	rs1051660	Yuferov, V. et al. [110]; Jones, J.D. et al. [111]; Nagaya, D. et al. [112]; Zhang, H. et al. [102]; Albonaim, A. et al. [113]; Gerra, G. et al. [114]; Kumar, D. et al. [115]; Levran, O. et al. [103].	Yuferov, V. et al. [110]; Jones, J.D. et al. [111]; Zhang, H. et al. [102]; Gerra, G. et al. [114]; Levran, O. et al. [103].		Yuferov, V. et al. [110]; Jones, J.D. et al. [111].	Yuferov, V. et al. [110]; Jones, J.D. et al. [111].
BDNF	rs6265; rs11030104; rs10767664; rs13306221; rs56164415; rs13306221; rs16917204; rs7127507; rs1967554; rs11030118; rs988748; rs2030324; rs11030119	Jin, T. et al. [ 116]; Jia, W. et al. [117]; Su, H. et al. [118]; de Cid, R. et al. [119].		Jin, T. et al. [116]; Jia, W. et al [117]; Su, H. et al. [118].		
NRXN3	rs10144398 rs10151731 rs10083466 rs1424850 rs221497 rs221473	Panagopoulos, V.N. et al. [120]; Lachman, H.M. et al. [121].	Panagopoulos, V.N. et al. [120]; Lachman, H.M. et al. [121].		Lachman, H.M. et al. [121].	Lachman, H.M. et al. [121].
COMT	rs4680	Henker, R.A. et al. [122];	Henker, R.A. et al. [122].	Vereczkei, A. et al. [90];		
		Vereczkei, A. et al. [90]; Li, T. et al. [123]; Rakvåg, T.T. et al. [124].		Li, T. et al. [123]; Rakvåg, T.T. et al. [124].		
SLC6A4	rs25531 rs1042173	Saiz, P.A. et al. [125]; Gerra, G. et al. [126]; Szilagyi, A. et al. [94]; Iamjan, S.A. et al. [127].	Gerra, G. et al. [126]; Szilagyi, A. et al. [94].		Saiz, P.A. et al. [125]; Iamjan, S.A. et al. [127].	
